# Does six months of structured group exercise improve physical activity, sedentary behavior, and sleep in people living with dementia? A pilot randomized controlled trial

**DOI:** 10.1002/alz.088549

**Published:** 2025-01-09

**Authors:** Olabamibo Oke, Yanbin Dong, Haidong Zhu, Andre Soares, Charmi Patel, Colleen Hergott, Jennifer L Waller, Lufei Young, Dawnchelle Robinson‐Johnson, Richard Sams, Mark Hamrick, Deborah A Jehu

**Affiliations:** ^1^ Augusta University, Augusta, GA USA; ^2^ University of North Carolina, Charlotte, NC USA; ^3^ Claiborne Assisted Living Facility, Augusta, GA USA; ^4^ Georgia War Veterans Nursing Home, Augusta, GA USA

## Abstract

**Background:**

People living with dementia (PWD) often have inactivity‐induced muscle atrophy, increased sedentary behavior, and circadian rhythm disorders. Exercise may improve physical activity, sedentary behavior, and sleep in PWD, but further research is needed. The purpose of this pilot randomized controlled trial (RCT) was to examine whether a structured exercise program improves physical activity, sedentary behavior, and sleep in PWD.

**Method:**

PWD were randomized (1:1) to exercise (n = 21) or usual care (n = 21) at two residential care facilities (NCT05488951; age = 82.1±8.05 years; Montreal Cognitive Assessment (MOCA) = 10.2 points; female = 35.7%; mobility device = 61.9%). Participants wore an actigraphy monitor on their non‐dominant wrist for 7 days (minimum of 4 days to be included in the analysis) at baseline and 6 months. The adapted Otago Exercise Program involved a physical therapist‐led strength, balance, and walking program for 1 hour, 3x/week for 6 months in groups of 5‐7. We controlled for age, race, sex, and the MOCA in the intent‐to‐treat (ITT; n = 42) and per protocol analyses (PPA; n = 21 usual care; n = 9 exercisers with 2/3 adherence).

**Result:**

The ITT analysis revealed that the exercise group (pre = 78.8%, post = 83.7%) had a greater percentage of sedentary activity than the usual care group (pre = 68.2%, post = 67.7%) at 6 months (p = 0.01). The PPA disclosed that the average length of sedentary bouts decreased in the usual care group (pre = 22.2, post = 20.1 minutes) compared to exercise group (pre = 46.9, post = 54.1 minutes) from baseline to 6 months (p = 0.01). Adherence to wearing the actigraphy monitor for ≥4 days was poor (pre: n = 25/42 PWD, post: n = 13/42 PWD).

**Conclusion:**

This RCT provides insufficient evidence to suggest that exercise impacts physical activity, sedentary behavior, or sleep among PWD. The results suggesting that usual care improves sedentary behavior over exercise should be interpreted with caution because this is a pilot RCT and adherence to wearing the actigraphy monitor was poor in both groups due to cognitive impairment. Future work is still needed to determine the effects of exercise on physical activity, sedentary behavior, and sleep, and should consider alternative placements of the actigraphy monitors, such as the back or lower leg, to increase adherence.

